# Physical activity motives, barriers, and preferences in people with obesity: A systematic review

**DOI:** 10.1371/journal.pone.0253114

**Published:** 2021-06-23

**Authors:** Aurélie Baillot, Stéphanie Chenail, Naiara Barros Polita, Mylène Simoneau, Mathilde Libourel, Evy Nazon, Eléonor Riesco, Dale S. Bond, Ahmed J. Romain

**Affiliations:** 1 Department of Nursing, University of Québec en Outaouais, Gatineau, QC, Canada; 2 Institut du savoir Montfort-Recherche, Ottawa, ON, Canada; 3 Centre de recherche du Centre Intégré de Santé et Services Sociaux de l’Outaouais, Gatineau, QC, Canada; 4 Department of Nursing, State University of Northern Paraná, Bandeirantes, PR, Brazil; 5 Faculty of Health Sciences, School of Human Kinetics, University of Ottawa, Ottawa, ON, Canada; 6 Faculty of Physical Activity Sciences, University of Sherbrooke and Research Center on Aging, CIUSSS de l’Estrie–CHUS, Sherbrooke, QC, Canada; 7 Institut des Sciences et Industries du vivant et de l’environnement, AgroParisTech, Paris, France; 8 Department of Psychiatry and Human Behavior, Weight Control and Diabetes Research Center, The Miriam Hospital/Brown Alpert Medical School, Providence, RI, United States of America; 9 Faculty of Medicine, School of Kinesiology and Physical Activity Sciences, Université de Montréal, Montréal, QC, Canada; 10 Centre de Recherche de l’Institut Universitaire en Santé Mentale de Montréal, Montréal, QC, Canada; University of South Australia, AUSTRALIA

## Abstract

**Background:**

Although the benefits of physical activity (PA) are well known, physical inactivity is highly prevalent among people with obesity. The objective of this systematic review was to i) appraise knowledge on PA motives, barriers, and preferences in individuals with obesity, and ii) quantify the most frequently reported PA motives, barriers and preferences in this population.

**Methods:**

Six databases (Pubmed, CINAHL, Psyarticle, SportDiscus, Web of science and Proquest) were searched by independent reviewers to identify relevant quantitative or qualitative articles reporting PA motives, barriers or preferences in adults with body mass index ≥ 30 kg/m^2^ (last searched in June 2020). Risk of bias for each study was assessed by two independent reviewers with the Mixed Methods Appraisal Tool (MMAT).

**Results:**

From 5,899 papers identified, a total of 27 studies, 14 quantitative, 10 qualitative and 3 mixed studies were included. About 30% of studies have a MMAT score below 50% (k = 8). The three most reported PA motives in people with obesity were weight management, energy/physical fitness, and social support. The three most common PA barriers were lack of self-discipline/motivation, pain or physical discomfort, and lack of time. Based on the only 4 studies available, walking seems to be the preferred mode of PA in people with obesity.

**Conclusions:**

Weight management, lack of motivation and pain are key PA motives and barriers in people with obesity, and should be addressed in future interventions to facilitate PA initiation and maintenance. Further research is needed to investigate the PA preferences of people with obesity.

## Introduction

Obesity is a major public health issue in North America affecting more than 25% of adults in Canada, and 40% of adults in the United States [1,2]. Obesity contributes to impaired physical and mental health-related quality of life, and increased morbidity and mortality [3,4]. Physical activity (PA) is a cornerstone of interdisciplinary obesity management [5,6]. Indeed, the benefits of regular PA on weight management, body composition, physical fitness, and cardiometabolic health in people with obesity are well documented [7–10]. However, more than half of Canadians and Americans living with obesity report to be insufficiently active [11,12]. Furthermore, adherence to structured PA interventions is poor and drop-out rates ranged from 20% to 80% [13].

In the context of PA intervention, a mismatch between patients’ preferences or motives, and the PA intervention planned could negatively impact PA engagement [14]. Integrating preferences into interventions has been considered as a patient-oriented strategy to improve participation and adherence as patients feel included in their decisions [14–17]. Moreover, from a PA perspective, previous research highlights that when people with obesity were offered to self-select their PA intensity, they accumulated more PA over time [18,19]. Along with PA preferences among individuals with obesity, it is important to understand PA motives and barriers to inform clinicians, and health stakeholders on the development of strategies to better improve PA behavior in this population [20–23].

Past systematic reviews on PA motives, barriers and preferences have been performed in various clinical populations (e.g., type 2 diabetes) [24–27]. Although several qualitative and quantitative studies are available on this topic in people with obesity, to our knowledge, none have systematically summarized this information. Only one systematic review of qualitative studies has been carried out on PA motives and barriers in people with severe obesity [28], and reported that weight loss was the main reason for exercising, followed by other motives, such as the risk of diseases, and skills improvement. Moreover, physical (e.g., health problems, weight, and pain), and psychosocial barriers (e.g., embarrassment, self-blame, lack of safety, and time) were also reported in this study [28].

Therefore, the objectives of the present systematic review were to i) appraise current qualitative and quantitative knowledge on PA motives, barriers and preferences in people with obesity, and ii) quantify which PA motives, barriers and preferences were more common in this population.

## Materials and methods

### Protocol and registration

The Preferred Reporting Items for Systematic Reviews and Meta-analysis (PRISMA) guidelines were used to perform this review [29]. The protocol was pre-registered in PROSPERO (CRD42020141447).

### Eligibility criteria

Quantitative and qualitative studies were included in this review if they met the following inclusion criteria: i) constituted primary research published in peer-reviewed journals with full-text available in English or French; ii) focused on adults (≥ 18 years old) with a body mass index (BMI) ≥ 30 kg/m^2^ (or more than 75% of the sample with BMI ≥ 30 kg∕m^2^ if the study did not exclusively include people with obesity or did not perform sub analysis in people with obesity); iii) reported motives, barriers or preferences to PA.

To define PA, the standard definition of Caspersen et al. (1985) and endorsed by the World Health Organization “any bodily movement produced by skeletal muscles that results in energy expenditure” was used in the present systematic review [30]. Motives were defined as any perceived reasons to increase and maintain PA, and barriers as any challenges reported by participants reducing PA initiation and maintenance [25]. Preferences were considered as patient-reported favourite choices concerning PA modalities, context, type, and supervision.

During the full-text papers selection, authors (AB, AJR, ER) decided by consensus to exclude studies that focused more on specific sub-populations with obesity, given that it would have not been possible to clearly distinguish whether PA motives, barriers, and preferences should be attributed to obesity or the coexistent condition/circumstance. These specific subpopulations included: pregnant women [31–33], cancers survivors [34–37], people with intellectual disabilities [38], veterans living with schizophrenia [39], and bariatric surgery patients [40–51].

### Information sources and search

A systematic search of eligible studies was conducted in six different databases (Pubmed, CINAHL, PsycArticles, SportDiscus, Web of science and Proquest). Reference lists from eligible studies, the 10 first pages of Google Scholar and Open Grey database, as well as personal records were checked to identify other potentially relevant studies (AB, AJR, DB).”

The search was performed on July 23, 2019 without date restriction, using research equation including keywords and Medical Subject Headings (Mesh) terms developed with a university librarian. For example, the PubMed search strategy was the following: "Exercise"[Mesh] AND "Obesity"[Mesh] AND ("Motivation"[Mesh] OR "Patient Preference "[Mesh] OR "preference*"[All Fields] OR "barrier*"[All Fields] OR "facilitator*"[All Fields]) OR "obstacle*"[All Fields]) AND "humans"[MeSH Terms] AND (English[lang] OR French[lang]) AND "adult"[MeSH Terms]. The search strategy was modified for each database, considering their specificities. An updated search was performed on June 04 (2020) to retrieve any potential studies published since the initial search. See supplemental material S1 File for detailed search strategies for each database. The search in Grey literature was performed on April 26, 2021.

### Study selection

All retrieved citations were imported into EndNote software (Version X9), and duplicate records were removed by one reviewer (ML). Two independent students’ reviewers (ML and MS) paired with senior reviewers (AB and AJR) screened records using a data extraction form against inclusion and exclusion criteria, first according to titles and abstracts, and then to the full-text papers of the selected abstracts. Disagreements were resolved by a third party (AB or AJR). If necessary, authors were contacted in case of missing or incomplete data for the study selection step.

### Data collection process

The following data were extracted by one review author (SC) using a data extraction form developed for the present review, and double-verified by two others (NBP, MS): authors; publication year; study setting; country; study design; sample size; participants’ characteristics (age, sex, BMI and comorbidities); methods to appraise PA motives, barriers and preferences; and results: survey/questionnaire items with the score or frequency associated for quantitative studies, and first and second constructs with adjectives reflecting the importance of the outcomes (many, several, etc.) for qualitative studies. To be extracted, PA motives and barriers should have been reported in the results section. Disagreements were resolved by having a fourth review author (AB) returning to the full text(s) to check the accuracy of extracted data.

### Data synthesis process

A thematic synthesis of the data extracted from qualitative studies was used following the steps proposed by Thomas and Harden [52]. First, line-by-line coding driven by the objectives was performed by one reviewer (NBP). Then, similar codes were grouped into descriptive themes, including first and second order constructs, which were verified by a second reviewer (SC). Analytical themes were generated by the interpretation of descriptive themes and validated by two authors (SC, AB). Finally, themes were compared and integrated to quantitative categories. Results on PA motives and barriers extracted from qualitative and quantitative studies were classified in three main categories: physical, psychological and socio-ecological by two reviewers (SC and NBP) [25], and then reviewer authors (AB, AJR, SC, NBP) created subcategories by consensus to merge similar items and constructs.

### Analyses

Regarding the statistical part, though a meta-analysis of proportion was planned to further rank each PA motives, barriers and preferences individually, this option was not found to be feasible given the small number of included studies per section and the high heterogeneity between included studies. Alternatively, based on the scale from Clifford et al. [24], we created a score of importance for each PA motive and barrier subcategory. Briefly, a score of importance ranging from 0 to 3 was assigned to each PA motive and barrier subcategory in each study (see Table 1 for details) by two independent reviewers (AB, SC). Disagreements were resolved during discussion with a third reviewer (AJR). For example, a score of 3 was assigned to pain as a PA barrier in studies reporting 50% or more of participants checked the item pain as a PA barrier. To then obtain a rank for each PA motive and barrier, a global score was calculated by summing each PA motive and barrier score across all the studies (maximal score of 33 for PA motives; 11 studies × 3, and 69 for PA barriers; 23 studies × 3).

**Table 1:** Physical activity barriers and motives score of importance based on the studies of Clifford et al. [24].

Scores	0	1	2*	3
**Quantitative data**				
Percentage of participants who checked item as a barrier (yes or no question)	PA barrier and motive not reported in the study	0–24%	25–49%	50–100%
Percentage of participants that agreed based on a Likert scale		0–24%	25–49%	50–100%
Percentage of participants who rated item as *major* barrier (responses ranged between 0.7 to 23.4%)		0–9%	10–19%	20–25%
Score: 5-point Likert (1 = strongly disagree, 3 = neither agree nor disagree, 5 = strongly agree)		1–2.0	2.1–3.0	3.1–5.0
Score. 5-point Likert scale, (1 = never, 2 = rarely, 3 = sometimes, 4 = often, or 5 = very often)		1–1.9	2.0–2.9	3.0–5.0
**Qualitative data**				
Adjectives	PA barrier and motive not reported in the study	A few women; Some participants; Several participants		Mentioned by all members; Commonly mentioned; Most mentioned; The first to third most common; Substantial barrier; Mentioned by many participants and as a significant barrier; Majority of participants; Prominent theme; Extreme barriers; One of the most expressed; Mentioned in all focus group

* A default score of 2 was assigned for barriers that were not able to be rated, but were reported.

For PA preferences, a narrative synthesis was favoured given the small number of studies (k = 4) and the high heterogeneity between studies.

### Risk of bias in individual studies

Risk of bias for each of the included studies was assessed by two independent reviewers (AB, SC) for quantitative studies, two other independent reviewers for qualitative studies (NBP, EN), and two independent reviewers for mixed studies (NR, AB) with the Mixed Methods Appraisal Tool (MMAT-Version11), adapted for this review. Any discrepancies were mediated by a third reviewer (AJR). Given the descriptive nature of our research objectives, the following criteria were used for quantitative studies: 1) Is the sampling strategy relevant to address the research question? 2) Is the sample representative of the target population? 3) Are the measurements appropriate? 4) Is the risk of nonresponse bias low? The 5 criteria for qualitative and mixed methods studies were those reported in the MMAT (respectively: 1. Is the qualitative approach appropriate to answer the research question? 2. Are the qualitative data collection methods adequate to address the research question? 3. Are the findings adequately derived from the data? 4. Is the interpretation of results sufficiently substantiated by data? 5. Is there coherence between qualitative data sources, collection, analysis and interpretation? / 1. Is there an adequate rationale for using a mixed method design to address the research question? 2. Are the qualitative data collection methods adequate to address the research question? 3. Are the outputs of the integration of qualitative and quantitative components adequately interpreted? 4. Are divergences and inconsistencies between quantitative and qualitative results adequately addressed? 5. Do the different components of the study adhere to the quality criteria of each tradition of the methods involved?) [53,54]. Each criterion was assessed as being fulfilled (1 point) or not fulfilled/insufficient information for adequate assessment (0 point), leading to a global score of 4 for quantitative studies, and 5 for qualitative and mixed studies. Scores were then converted to percentage to facilitate between-studies comparison.

## Results

### Study selection

**Fig 1:**
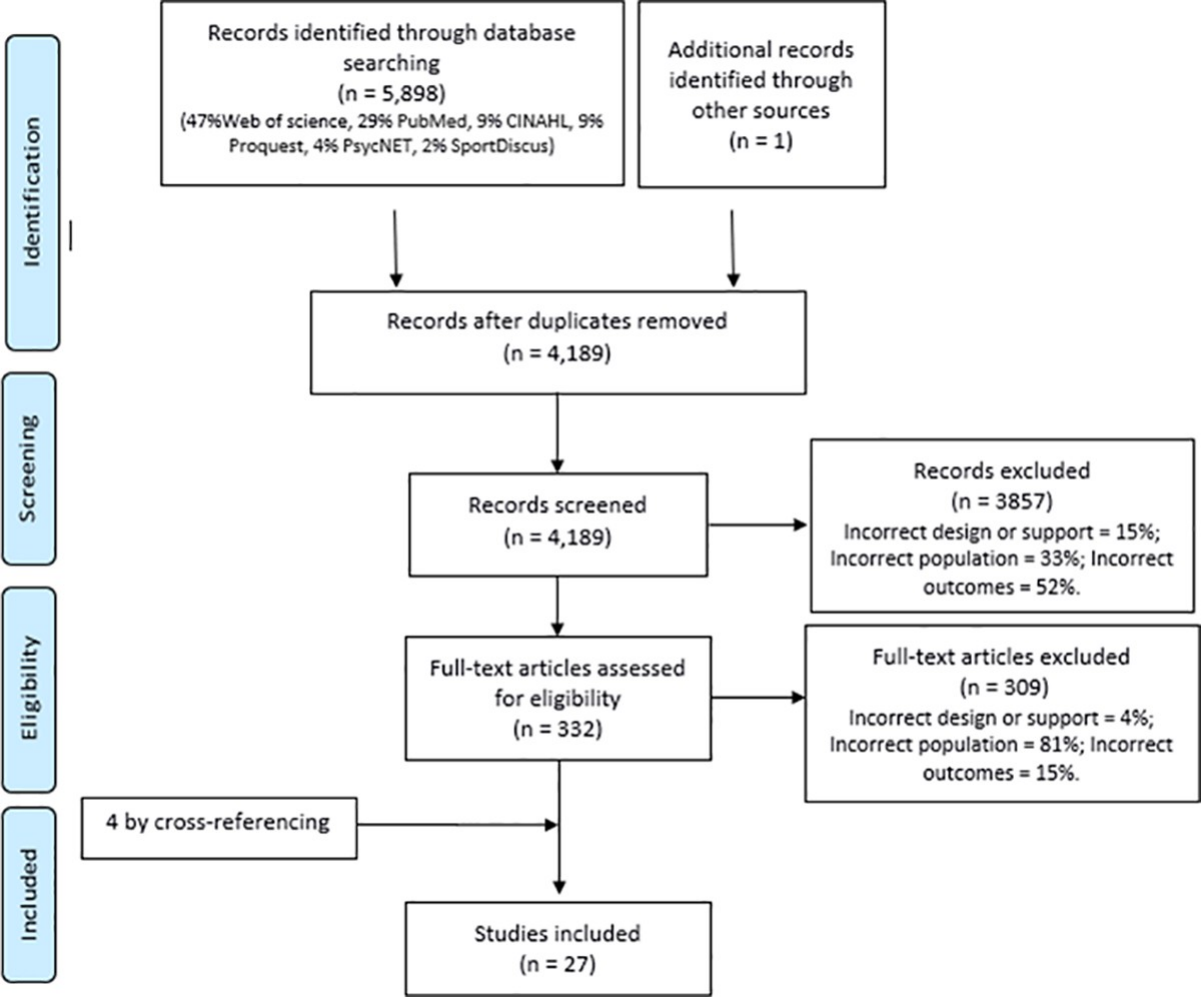
PRISMA flow diagram.

The electronic searches generated 5,899 studies; which was reduced to 4,189 after removing duplicates. Following this step, 3,857 records were excluded based on title and abstract, with 52% of them because they did not present the outcomes of interest. Twenty-seven studies in total were included in this review (Fig 1).

### Study characteristics

Table 2 describes studies and participants’ characteristics. Briefly, 14 quantitative studies (51.9%) [55–68], 10 qualitative studies (37.0%) [69–78], and 3 mixed studies (11.1%) [79–81] were included. Among quantitative studies, 11 studies were observational [55–59,63–68], and 3 were interventional studies [60–62]. However, all interventional studies reported information about barriers and/or motives to habitual PA, not about PA intervention. Almost half of the studies were performed in the United States (k = 13) [56,58,59,61–63,65,69,70,74,79–81], and 37.0% published in the last five years (2015–2020; k = 10) [55,56,65,68–71,74,75,79]. A total of 8,065 participants with obesity were assessed within the included studies (k = 27), with a median age of 46 years old (range 37.8–62.0) (k = 10 because information is missing for people with obesity in 17 studies). More than half of the studies included over 70% of women in their sample (51.9%; k = 14) [56,58,59,61–63,65,69,72,74,77,79–81], 37.0% included only women (k = 10) [56,58,61–63,69,74,77,79,81], and 7.4% included only men (k = 2) [55,78].

**Table 2:** Characteristics of the studies included (k = 26).

(Reference) *Publication Year* (Country)	Sample size (% of women)	People with obesity *[all sample data]*			Comorbidities (%) and specific characteristics	Outcomes assessed**	Methods/Tools	MMAT scores***
		%	Age years±SD or (range)	BMI kg/m^2^±SD or (range)				
**Quantitative studies**								
Ashton [55] *2017* (Australia)	282 (0%)	10%	NR *[22*.*3±2*.*1]*	NR *[24*.*7±4*.*4]*	NR	Barriers Motives	Online questionnaire	25
Masterson [56] *2017* (USA)	630 (100%)	41%	49.7±15.4	NR	T2D (30.6%), HBP (25.7%) Mild (15.8%) and moderate-severe depression (26.1%) Urban Latinas	Barriers	5 questions the Influences on PA Instrument about barriers to PA	25
Egan [57] *2013* (Ireland)	145 (36%)	100%	59.0±11.0	34.0 (IQR: 32.0–37.5)	T2D (100%)	Barriers	List with scale	0
Genkinger [58] *2006* (USA)	120 (100%)	65%	NR *[48*.*0±11*.*0]*	NR	NR African American in church-based, PA intervention	Barriers	Adapted version of Steinhardt/Dishman Barriers for Habitual PA Scale	50
James [59] *2008* (USA)	823 (71%)	41%	Class I: 51.0 (19–84) Class II: 48.3 (18–87)	NR	NR African American and part of a colorectal cancer prevention intervention churches rural	Barriers	Phone questionnaire	50
Labrunee [60] *2012* (France)	23 (57%)	100%	52.8±8.5	40.1± 7.3 (control) 39.3±9.9 (intervention)	Diabetes (100%) Enrolled in PA intervention	Barriers Preferences	Phone questionnaire	0
Napolitano [61] *2011* (USA)	280 (100%)	38%	NR *[47*.*3±10*.*7]*	NR *[28*.*7±5*.*2]*	NR Previously inactive women	Barriers	Expected outcomes and barriers for habitual PA scale	25
Rimmer [62] *2010* (USA)	33 (100%)	91%	NR *[60*.*1 ± 10*.*1]*	NR *[49*.*1±12*.*4]*	Arthritis (67%), Multiple sclerosis (6%), Stroke (6%), Back problem (6%) Sedentary African-American	Barriers	Barriers to PA Questionnaire for People with Disabilities	25
Rye [63] *2009* (USA)	702 (100%)	60%	NR *[52*.*2±6*.*8]*	NR	NR White, low-income women	Barriers	Questionnaire	50
Skov-Ettrup [64] *2014* (Danemark)	55655 (61%)	6%	NR	NR	NR PA during the past year	Motives	Internet or paper-based questionnaire	25
Stankevitz [65] *2017* (USA)	124 (93%)	100%	45.0±9.0	37.7± 6.7	NR	Barriers	Internet or mail questionnaire	25
Burton [66] *2012* (Australia)	7784 (56%)	23%	NR *[(42–67)]*	NR	NR	Preferences	Mail questionnaire	75
Short [67] *2014* (Australia)	1137 (50%)	30%	*[52*.*8±16*.*3]*	*[30*.*0±14*.*7]*	Chronic illness (46%)	Preferences	Phone questionnaire	50
Borodulin [68] *2016* (Finland)	2260 (59%)	20%	NR *[(18–64)]*	NR	NR	Barriers	Questionnaire	75
**Qualitative studies**								
Bowen [69] *2015* (USA)	9 (100%)	78%	NR *[*75.0±5.3*]*	NR *[*27–41*]*	67% reported using canes or walkers	Barriers Motives	Semi-structured interviews	100
Coe [70] *2017* (USA)	13 (54%)	100%	42.0 (29–53)	52.5 (37–81)	100% at least one comorbidity (HBP, dyslipidemia, or T2D) African American	Barriers	Focus group	100
Danielsen [71] *2016* (Norway)*	8 (63%)	100%	NR (35–63)	NR (37–60)	Part of a 10–14 weeks inpatient lifestyle modification program	Barriers Motives	Interviews	100
Guess [72] *2012* (UK)	29 (83%)	21%	37.8±10.9	46.8±5.6	Attending dietetic clinics for weight management	Barriers Motives	Focus groups and semi-structured interviews	60
Igelström [73] *2012* (Sweden)*	15 (47%)	100%	62.0 (IQR 8.5)	37.0 (IQR 5.0)	Obstructive sleep apnea and CPAP treatment (100%)	Barriers Motives	Semi-structured interviews	100
Joseph [74] *2017* (USA)	25 (100%)	100%	38.5±7.8	39.4±7.3	Sedentary lifestyle (100%)	Barriers Motives Preferences	Focus group	100
Lidegaard [75] *2016* (Denmark)	28 (46%)	86%	NR *[59*.*4±8*.*8]*	NR *[34*.*4±5*.*0]*	T2D (100%); Heart disease or musculoskeletal disorders (79%)	Barriers Motives	Focus group + probes in the form of images, statements and quotations regarding PA.	100
Piana [76] *2013* (Italy)	80 (63%)	100%	53.2±12.2	36.5±5.9	HBP (49%); T2D (35%) Coronary Heart Disease (4%)	Barriers	Focus group	100
Groven [77] *2010* (Norway)	5 (100%)	100%	NR (35–63)	NR (40–48)	Part of a weight loss program	Barriers Motives	Semi-structured interviews	100
Lewis [78] *2011* (Australia)	36 (0%)	100%	46.0 (21–69)	37.1 (30–61)	HBP (33.3%); Arthritis and joint problems (30.6%); Sleep apnea (16.7%); Diabetes (13.9%), Cardiovascular problems (11.1%)	Barriers Motives	Interviews	60
**Mixed-methods studies**								
Adachi-Mejia [79] *2016* (USA)*	78 (100%)	76%	NR *[52*.*8±14*.*5]*	NR *[35*.*4±9*.*2]*	NR Enrolled in a lifestyle community-based program for vulnerable populations	Barriers	Survey + one open question	80
Lattimore [80] *2011* (USA)	384 (78%)	46%	NR *[50–75+]*	NR	NR Adults 50 years and older	Barriers	Interviews	80
Leone [81] *2013* (USA)	Quanti 195 (100%)	51%	NR *[55*.*7±7*.*0]*	NR *[35*.*7±7*.*0]*	NR Inactive white women over 50 years old	Barriers Motives	Online survey	80
	19 (100%)	100%	55 (50–72)	36.0 (28.2–46.6)			Focus group	

* when country of recruitment was not reported country of the authors is reported

** = only outcomes assessed in people with obesity were reported in this column

*** = MMAT scores are expressed as a % of the maximum possible score; BMI = body mass index; CPAP = Continuous positive air pressure; HBP = High blood pressure; MMAT = Mixed Methods Appraisal Tool; NR = not reported; PA = physical activity; T2D = type 2 diabetes.

Regarding the outcomes of interest, PA barriers were assessed in 24 studies (88.9%) [11 quantitative, 10 qualitative, and 3 mixed studies] [55–63,65,68–81], motives in 11 studies (40.7%) [2 quantitative, 8 qualitative, and 1 mixed studies] [55,64,69,71–75,77,78,81], and preferences in 4 studies (14.8%) [3 quantitative and 1 qualitative studies] [60,66,67,74].

### Risk of bias

More than half of the quantitative studies have a MMAT score below 50% (57.1%, k = 8) [55–57,60–62,64,65,68], and all qualitative and mixed studies had a score above 50% (see Table 2 and supplemental S1 Table). The scores of the quantitative studies below 50% are explained by the fact that non-probability sampling was performed in these studies, impacting the representativeness of the sample and our ability to know the nonresponse rate.

### Findings for physical activity motives

**Fig 2:**
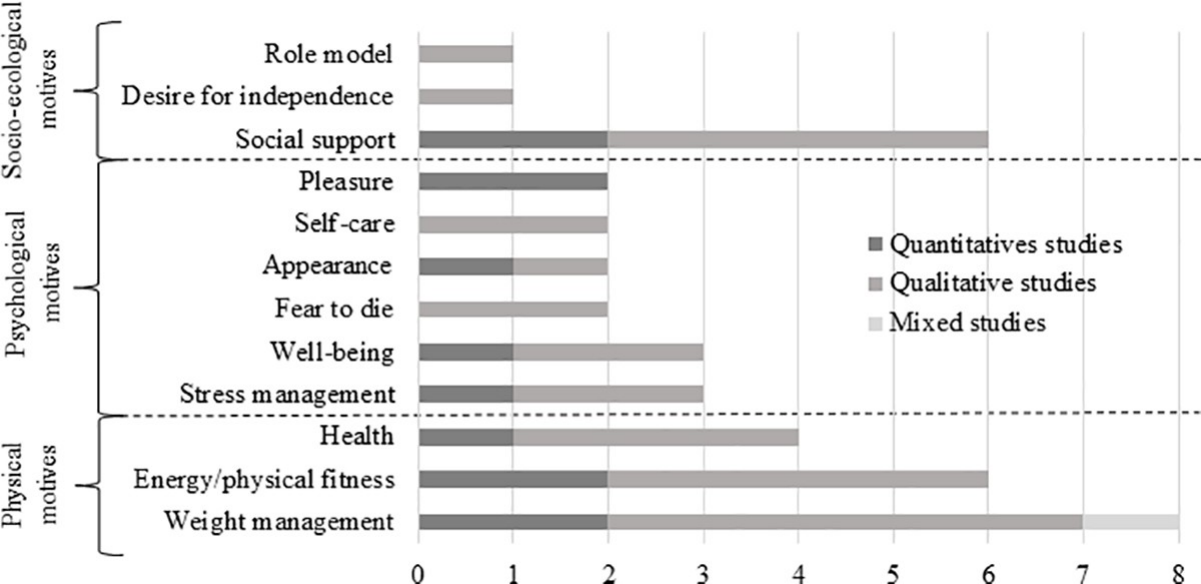
Number of studies according to each categories of physical activity motives in people with obesity.

**Fig 3:**
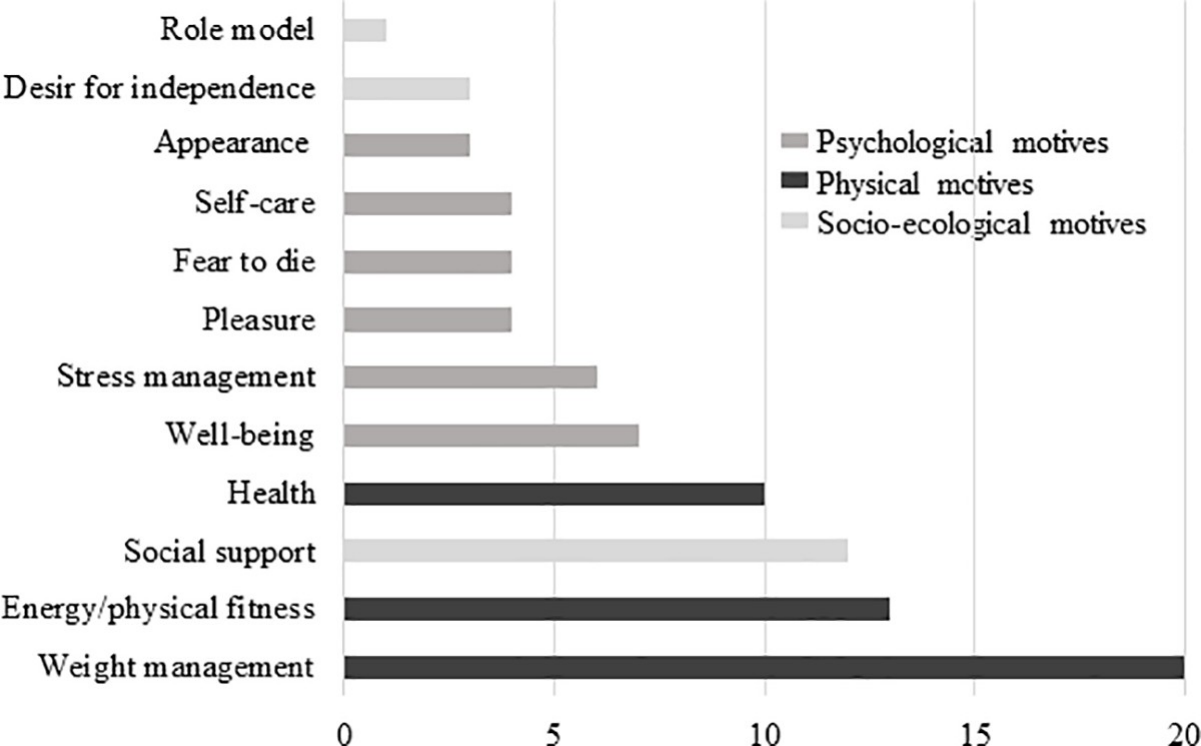
Importance scores of motives to physical activity in people with obesity.

The 12 PA motives identified were classified in different categories of motives: 6 psychosocial (50.0%), 3 socio-ecological (25.0%) and 3 physical motives (25.0%) (Fig 2 and supplemental data S2 Table). More than half (k = 7/12, 58.3%) of PA motives comes from both quantitative and qualitative studies, 33.3% (k = 4/12) from qualitative studies only, and 8.3% (k = 1/12) from quantitative study (Fig 2). In the different included studies, the three most reported PA motives among studies were weight management (k = 8/11, 72.7%), energy/physical fitness (k = 6/11, 54.5%), and social support (k = 6/11, 54.5%) (Fig 2). Regarding PA motives in terms of ranking, the three with the highest scores of importance were also weight management (score = 20), energy/physical fitness (score = 13) and social support (score = 12) (Fig 3).

### Findings for physical activity barriers

**Fig 4:**
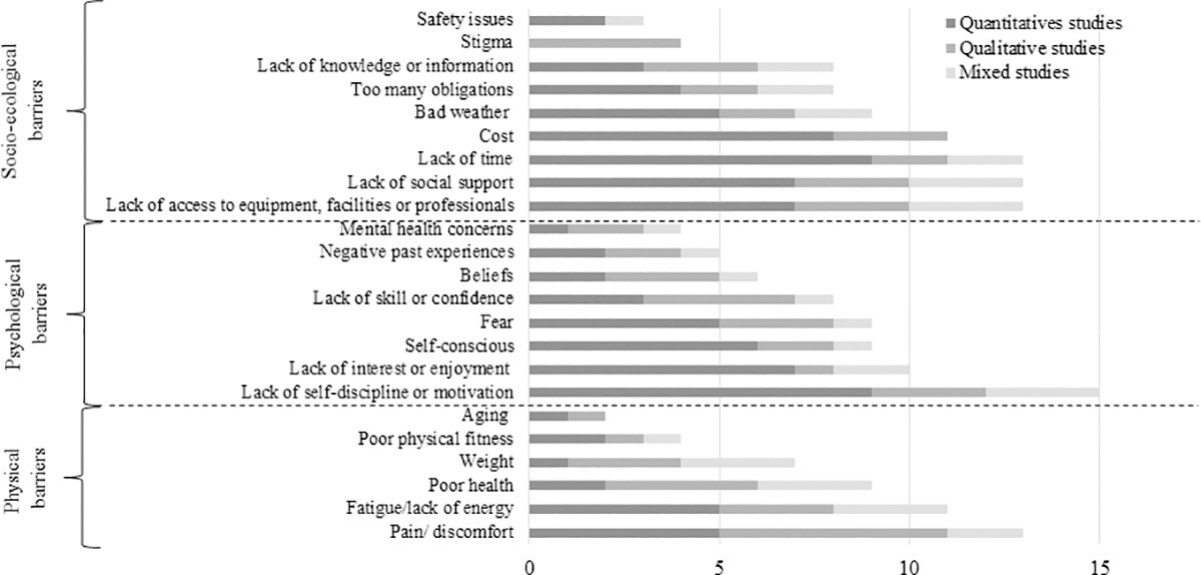
Number of studies according to each categories of physical activity barriers in people with obesity.

Barriers were classified in three categories being socio-ecological barriers (k = 9; 39.1%), psychological barriers (k = 8; 34.8%), and physical barriers (k = 6; 26.1%) (Fig 4 and supplemental data S3 Table). Except for stigma (only qualitative studies), each PA barrier was studied in both quantitative and qualitative studies, and the most reported PA barriers among studies being lack of self-discipline/motivation (k = 15/24, 62.5%), pain/ physical discomfort (k = 13/24, 54.2%), lack of time (k = 13/24, 54.2%), lack of social support (k = 13/24, 54.2%) and lack of access to equipment, facilities or professionals (k = 13/24, 54.2%) (Fig 4).

**Fig 5:**
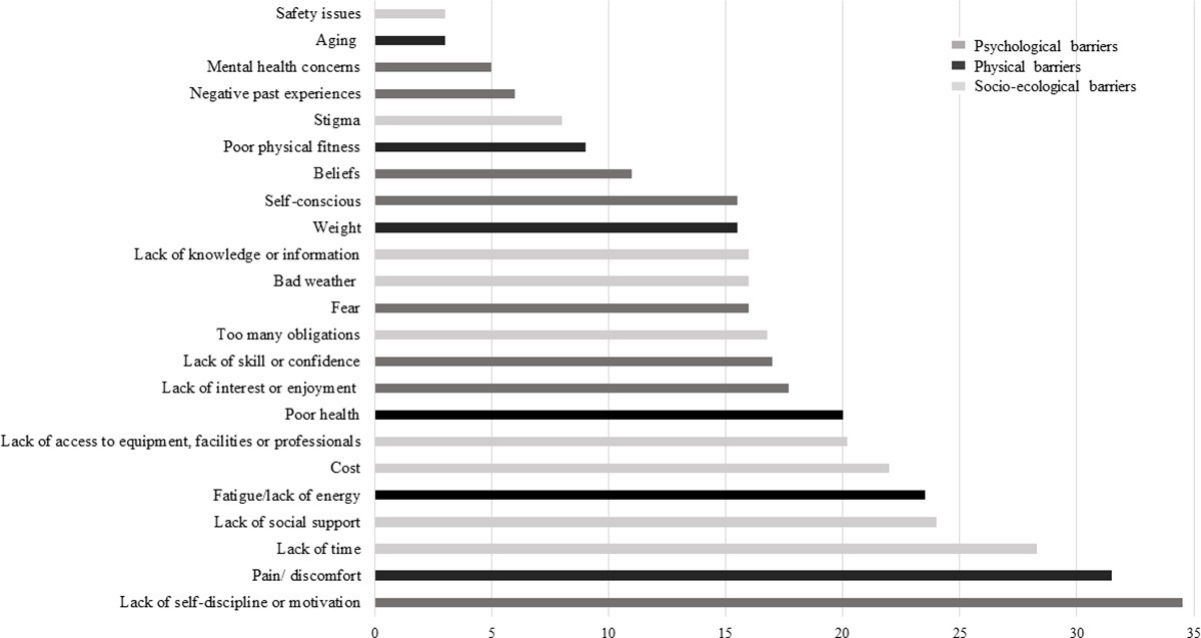
Importance scores of barriers to physical activity in people with obesity.

The top three physical barriers to PA with the highest score of importance were pain/physical discomfort (score = 31.5), fatigue/lack of energy (score = 23.5) and poor health (score = 20) (Fig 5). Regarding psychological barriers to PA, lack of self-discipline/motivation (score = 34.5), lack of interest/enjoyment (score = 17.7), and lack of skills/confidence (score = 17.0) were the most frequently reported (Fig 5). For socio-ecological barriers to PA, lack of time (score = 28.3), lack of social support (score = 24.0) and cost (score = 22.0) were the three barriers with the highest score of importance (Fig 5).

### Findings for physical activity preferences

Among the four studies reporting results on PA preferences in people with obesity, two provided information on PA preferences (50%) [60,74], one on preferred PA context (25%) [66], and one on preferred PA delivery mode for intervention (25%) [67].

Only, Labrunée et al. (2012) and Joseph et al. (2017) provided data on PA type preferences in people with obesity (n = 23 and 25, respectively). Labrunée et al. (2012) asked to classify PA by order of preference the type of physical activity, while Joseph et al. (2017) asked the following open question: “What type of physical activity or exercise do you enjoy doing?”. Among people with obesity walking was the most preferred/enjoyed activity for both men and women [60,74]. Otherwise, the other preferred PA were cycling, swimming and rowing in the Labrunée et al. (2012) study [60], and dance or Zumba, cycling, water activities, and martial arts in the Joseph et al. (2017) study which was performed among women with obesity [74]. Resistance training was less often identified as a preferred type of PA [74].

Regarding the preferred PA context, Burton et al. (2012) examined it among 1781 middle-aged adults with obesity [66] by asking them to indicate their agreement, neutrality (no preference) or disagreement with a preference for each PA context. Results showed that 78% of participants preferred low-cost or no-cost PA, 61% preferred PA interventions that are not just about exercise, and 50% opted for PA with a format/routine set. Moreover, 26%, 15%, and 16% of participants, respectively, preferred supervised, team-based and competitive PA. Nearly a quarter (23%) of participants reported a preference toward PA requiring skills and practice, and less than half of participants preferred vigorous PA (30%) done at a fixed schedule time (43%). In terms of location, 86% of participants reported a preference for PA that can be done close to home, and 59% preferred outdoor activities. Regarding social settings, 73% of individuals indicated a preference towards activities that can be performed alone, 49% preferred activities with people around their age and 28% with individuals with same sex.

Short et al. (2014) analyzed the preferred PA intervention delivery mode, and provided the prevalence of people with obesity in four groups of people according to their most preferred mode of delivery (n = 1137 with 341 people with obesity): face-to-face program with an instructor (36% of survey responders including 35% of people with obesity), group-based program (44% of survey responders including 27% of people with obesity), program that can be done on their own using mailed and printed materials (11% of survey responders including 26% of people with obesity), programs that can be done on their own using internet (9% of survey responders including 24% of people with obesity) [67].

### Comparison between BMI classes

Among the currently available studies (k = 27), only a few have investigated whether PA motives (k = 2), barriers (k = 6) and preferences (k = 2) differed among individuals with and without obesity. As shown in Table 3, weight management (k = 2/2), was the only motive that differed across BMI classes [55,64]. Studies reported that weight management was a more prevalent PA motivator in adults with obesity compared to adults without obesity [55,64]. Regarding PA barriers, though no difference between BMI classes were noted in socio-ecological barriers, weight (k = 3/3; [61,79,81]), lack of self-discipline/motivation (k = 4/5; [59,61,68,79,80]) and self-consciousness (k = 3/3; [59,61,81] were more frequently reported by individuals with obesity than those without obesity.

**Table 3:** Physical activity motives and barriers comparison across body mass index classes (k = 7).

**Motives**	**-**	**0**	**+**	**++**
**Physical factors (k = 2)**				
Weight management (k = 2)			[64]	[55]
Energy/physical fitness (k = 2)		[55]	[64]	
Health (k = 2)		[55]		
**Psychological factors (k = 2)**				
Fear to die/live longer (k = 1)		[55]		
Well-being (k = 1)		[55]		
Appearance (k = 1)		[55]		
Pleasure (k = 2)		[55, 64]		
Stress management (k = 1)	[64]*			
**Socio-ecological factors (k = 2)**				
Socialize (k = 1)		[64]		
Social influence (k = 1)		[55]		
**Barriers**	**-**	**0**	**+**	**++**
**Physical factors (k = 4)**				
Poor health (k = 4)		[61, 80]		[79, 81]
Weight (k = 3)				[61, 79, 81]
Pain (k = 1)				[61]
Fatigue/lack of energy (k = 4)	[79]	[61, 80, 81]		
**Psychological factors (k = 6)**				
Fear (k = 2)		[61]		[79]
Lack of self-discipline/motivation (k = 5)		[68, 80]		[59, 61, 68, 79]
Lack of interest/enjoyment (k = 3)		[61, 80]		[81]
Mental health concerns (k = 1)				[79]
Lack of skills (k = 1)		[61]		
Self-consciousness (k = 3)				[59, 61, 81]
Negative past-experience (k = 1)		[80]		
Beliefs (k = 1)				[81]
**Socio-ecological factors (k = 6)**				
Lack of time (k = 5)	[68, 80]	[61, 68, 79]	[56]	
Too many obligations (k = 2)		[56, 61]		
Lack of social support (k = 5)	[80]	[61, 68, 79, 81]		[68]
Bad weather (k = 3)		[61, 79, 80]		
Lack of knowledge/information (k = 1)		[61]		
Cost (k = 3)		[61, 68]	[56]	
Poor access to facilities (k = 4)		[61, 80, 81]		[56]

- = less reported in people with obesity compared to normal and overweight people; 0 = no difference between BMI classes¸ + = more reported in people with overweight and obesity compared to normal weight people, ++ = more reported in people with obesity compared to normal and overweight people

* Compared to normal weight adults only

** = in men with obesity

^#^ = in women with obesity.

Based on the two studies that have investigated associations between BMI or obesity with PA preferences [66,67], social context seems particularly important among individuals with obesity. In fact, supervised [66], face-to-face [67] intervention was preferred to group-based intervention. However, in a context of group-based intervention, Burton et al. (2011) reported that homogeneous groups in terms of age and sex were preferred for individuals with a BMI greater than 30 kg/m^2^ [66].

## Discussion

The objective of the present study was to investigate PA motives, barriers and preferences in people with obesity. To our knowledge, the present review is the first to systematically address these questions in this population. From the studies (i.e. 14 quantitative, 10 qualitative, and 3 mixed methods) included in this review, 48.2% were performed in the United States with a clear predominance of women participants. Barriers to PA were most frequently investigated (k = 24) followed by PA motives (k = 11), and preferences to PA (k = 4).

The three **most common PA motives** reported by people with obesity, based on the scale of Clifford et al. [24], were weight management, energy/physical fitness and social support.

Unsurprisingly, **weight management** was the most frequently reported motive for PA in people with obesity, in accordance with previous qualitative review in people with severe obesity [28]. In addition, weight management is the only motive in individuals with obesity, which differs significantly from adults without obesity according to our literature review [55,64,81] (Table 3). This motive is an important factor for health professionals to consider when developing and implementing PA interventions. Indeed, previous studies showed that PA alone produces only modest weight loss [7], and could therefore lead to PA discontinuation. So, to facilitate PA over time, people should be informed that in a weight management context, PA has a more important role in terms of weight loss maintenance or waist circumference reduction, [6,7].

The second most frequently reported PA motive in people with obesity was **physical fitness improvement**. This motive is relevant given that an improvement of physical fitness can be achieved through PA interventions in people with obesity [6]. In addition, previous studies support the importance of physical fitness by showing that physically fit people with obesity have a reduced rate of all-cause mortality compared to unfit people with and without obesity [82]. However, weight and low physical fitness are also PA barriers in people with obesity, as shown in our results (Figs 4 and 5). Consequently, these barriers should be addressed and previous studies showed that PA counseling, and intervention tailored to physical fitness and weight could improve them [83,84].

According to our literature review, **key PA barriers** were lack of motivation/self-discipline, pain/physical discomfort, and lack of time in people with obesity.

**Low motivation and lack of time** are non-weight related PA barriers prominent in non-clinical and clinical populations [24,48,85,86]. However, studies included in our review seem to indicate that lack of self-discipline/motivation is more often reported in people with obesity (Table 3). Behavioral interventions including motivational interviewing are effective options to address lack of motivation, given its efficacy to improve PA adherence [87]. Regarding lack of time to exercise, though often underlined, several time-use studies highlighted that this PA barrier is more likely to reflect a low priority attributed to PA compared to other activities [86,88]. Moreover, people who have free time are not more active, thus simply helping them to find time for PA in their day might not improve PA in people with obesity [86]. It could be beneficial to implement behavioural interventions in people with obesity to support them to find motivation to change PA habits in this context. Several time-efficient solutions can be proposed like PA during transportation, PA during work break, reducing TV viewing, [86,87]. As a response to lack of time, it may be tempting to recommend high-intensity interval training to overcome time barriers, given the assumption that with higher intensity, exercise duration can be reduced, and seems equally effective to reduce fat mass and more effective to increase physical fitness in people with obesity compared with moderate-intensity continuous training [89–91]. Nevertheless, there is a debate regarding the relevance of high- compared to moderate-intensity exercise in adults living with obesity to increase long-term PA levels [92]. Indeed, knowing that adults living with obesity 1) avoid vigorous-intensity PA [93,94], and 2) are willing to accept longer exercise durations if the intensity remains low [95] emphasizing high intensity could be counter productive.

Regarding **pain**, the second most frequently reported PA barriers, people with obesity are more likely to suffer from pain [96,97], explaining why it is an important barrier to PA in this population, consistently put forward in other studies [98], and also compared to other BMI classes [61]. Previous studies hypothesized a bidirectional association between pain and PA in people with obesity. Musculoskeletal pain, the main source of pain described in qualitative studies can act as a functional limitation to engage and maintain PA [97]. At the same time, regular PA can reduce chronic musculoskeletal pain in people with obesity, due to its potential positive impacts on inflammation, psychological outcomes (e.g., mood, pain catastrophizing, etc.), muscle strength and coordination [97]. Nevertheless, PA for pain management in people with obesity requires support to tailor its practice, safety and efficacy [97]. The adjustment of PA volume (duration, intensity, frequency), joint range of motion during exercise, as well as the type of PA (non-impact PA) are valuable strategies that can be used for the pain management in order to increase PA adherence [97].

**Weigh**t is also a major physical obstacle to PA in people with obesity compared to people without obesity [61,79,81]. Interestingly, qualitative data from a study included in this review [81] revealed that women tend to perform more exercise when they lose weight because doing so gets easier. However, weight loss cannot be considered as facilitator *per se* given previous studies underlined that even after a massive weight loss, people remain physically inactive [98,99].

No conclusion on **PA preferences** can be drawn due to the small number of studies, and the different assessment of preferences (context vs. mode of delivery). However, walking seems to be preferred by people with obesity, as in the general North American population [100,101], probably because walking does not require any specific skill, equipment or place, and can be integrated easily into everyday life [102]. In addition, walking interventions are feasible and effective to improve the health among people with obesity [103]. Hence, regular walking can be proposed by health professionals as an option in the management of obesity and inactivity.

Considering the comparison between BMI classes, unfortunately, the paucity of data regarding how obesity classes affect PA preferences strongly limits generalization [66,67]. Nevertheless, some reflections emerged from this review and may be considered when PA recommendations are provided. Indeed, it appears that supervised [66] and individual [67] PA is preferred among individuals with obesity compared to their counterparts without obesity.

Finally, while there is still inconsistency about the interest in group-based PA [66,67], it seems that exercising with people of the same age and sex may be of importance for adults living with obesity. This suggests that feeling emotionally secure and socially accepted should not be underestimated [104]. This is consistent with the fact that self-consciousness, a psychological barrier related to self-image and embarrassment during exercise, is a major PA barrier in adults living with obesity compared to other BMI classes (Table 3).

In addition, people with obesity declared that socialization, group belonging, family, professional or peer support motive them to engage, perform and maintain PA practice (S2 Table). This result is in accordance with several previous studies showing positive associations between social support with PA attendance and adherence [105,106]. Nevertheless, additional studies are necessary in people with obesity, due to inverse results (no or negative association) to better understand the complex relationship between PA and social support [105,106]. Indeed, social support can be perceived as a PA barrier or motive according to people or context of practice, resulting in PA avoidance and isolation or PA adherence and socialization [28]. Hence, it may be relevant to work on public health messages to successfully promote PA and favour a lower obesity stigmatization by providing a better training to health professionals [107].

The main strength of the present review is the systematic inclusion of both qualitative and quantitative literature, allowing a larger integration of PA barriers, motives, and preferences of people with obesity. However, some limitations should be considered to better interpret data. First, only English and French full texts have been included. A second limitation is the characteristics of the included studies as half of studies comes from the United States, and all from occidental countries, and men with obesity are underrepresented. In addition, socioeconomic data of people with obesity are often missing in the included studies, not reported or only reported in all the sample, including people with and without obesity. However, it should be considered that ethnic diversity within the included studies is quite present, with 5 (19.2%) studies performed in ethnic minority groups (Urban Latinas, and African American) [56,58,59,62,70]. Third, the use of a scale to quantify the importance of each barrier and motive is not the most accurate method compared to meta-analysis, but allowed in this context the integration of quantitative and qualitative studies, as well as to compare quantitative results with different kinds of questions (score vs. prevalence, Likert scale vs. yes/no answers). In addition, especially for PA motives, the relevance of this score is limited due to the high number of missing data (score 2 was attributed to more than 60% of the PA motives). Fourth, the change of exclusion criteria during the full-text selection could have introduced bias. However, this choice was made to avoid the capture of barriers, motives and preferences reflecting more the specific condition/circumstance than obesity. Finally, publication bias could also affect our findings.

Based on identified gaps in the literature, future research should focus on more representative sample of people with obesity. To date, most of the studies were conducted with women in occidental countries, and several included quantitative studies have selection bias. A strong need to determine PA preferences in people with obesity has been also identified, given only four studies are currently available. In addition, the use of a common unit (e.g., percentage rather than score) or the development of validated questionnaire in people with obesity could be useful to harmonize results and obtain better idea of the importance of each PA motives, barriers and preferences. Otherwise, self-consciousness, an important barrier in people with obesity (Table 3), as well as stigmatization which was considered only in qualitative study according to our findings (Fig 4) should be systematically considered in future studies. Finally, there is also a need to consider PA motives, barriers, and preferences differences according to gender, age, socioeconomic status, health status, PA level to better address diversity and specific needs. Indeed, differences between sexes have been already shown in people with overweight, with women reported more often being too fat, embarrassed, and with not good enough health as a PA barrier compared to men [108].

To conclude, weight management, lack of motivation and pain are important PA motives and barriers in people with obesity. PA motives and barriers are both weight and non-weight related in people with obesity. For this reason, weight loss cannot be the only solution to remove PA barriers, and these should be addressed in PA interventions with the support of health professionals to facilitate PA initiation and maintenance. Further research is needed to investigate the PA preferences of people with obesity. Although, one size intervention does not fit all, the improvement of knowledge on PA barriers, motives and preferences would help health professionals to better address them, and develop intervention to reach the larger number of people with obesity in order to decrease physical inactivity in this population.

## Supporting information

S1 Checklist PRISMA 2009 checklist. (DOC)

S1 File Search equation. (DOCX)

S1 Table Risk of bias assessed with the Mixed Methods Appraisal Tool. (DOCX)

S2 Table Data extraction and classification details for physical activity motives. (DOCX)

S3 Table Data extraction and classification details for physical activity barriers. (DOCX)
