# Supplementary material for: Physical activity motives, barriers, and preferences in people with obesity: A systematic review
Source: PLoS One. 2021 Jun 23;16(6):e0253114. doi: 10.1371/journal.pone.0253114 (PMC8221526; doi:10.1371/journal.pone.0253114)
Supplement: S1 File — (DOCX) [file pone.0253114.s002.docx]

**PUBMED with MesH-terms**

(((Exercise[MeSH Terms]) AND (((((Motiv*) OR preference*) OR barrier*) OR facilitator*) OR obstacle*)) AND Obesity[MeSH Terms]) AND humans [MeSH Terms]

Limits: humans / English or French / adult 19+ years

**PUBMED with keywords**

(Exercise*[All Fields] OR Endurance [All Fields] OR Aerobic* [All Fields] OR Resistance adj1(activit* or train*)[All Fields] OR Muscl* adj2 (exercis* or strength) [All Fields] OR Sport [All Fields] OR Physical adj3 (activit* or train* or therap* or rehabili*) [All Fields] OR Relaxation [All Fields] OR Flexibility [All Fields] OR Stretch* [All Fields] OR Walk*[All Fields] OR Yoga[All Fields]) AND (obes*[Title/Abstract]) AND (motiv*[All Fields] OR preference*[All Fields] OR barrier*[All Fields] OR facilitator*[All Fields] OR obstacle*[All Fields])

Filters : English/French; Adult:19+; Humans

**Cinahl**

(MH "Obesity") AND ((MH "Exercise") OR (MH "Physical Education and Training") OR (MH "Physical Fitness") OR (MH "Physical Activity") OR (MH "Therapeutic Exercise") OR (MH "Yoga") OR (MH "Dance Therapy") OR (MH "Tai Chi") OR (MH "Sports")) AND ((TX "Motiv*") OR (TX preference*) OR (TX barrier*) OR (TX facilitator*) OR (TX obstacle*))

Opérateurs de restriction : All Adult; Peer Reviewed; English, French

**PsycNET**

((**Any Field**: (Weightlifting)) *OR* (**Any Field**: (Physical Activity)) *OR*(**Index Terms**: (Aerobic Exercise)) *OR* (**Index Terms**: (exercise)) *OR* (**Any Field**: (Yoga))) *AND* ((**Index Terms**: (obesity))) *AND* ((**Any Field**: (motiv*)) *OR* (**Any Field**: (obstacle*)) *OR* (**Any Field**: (preference*)) *OR* (**Any Field**: (barrier*)) *AND* (**Any Field**: (facilitator*))) *AND* **Publication Type**: Peer Reviewed Journal *AND* **Age Group**: Adulthood (18 yrs & older)

**Sportdiscus**

SU "OBESITY" AND SU "EXERCISE" AND (motiv* OR preference* OR barrier* OR facilitator* OR obstacle*)

Limiters: Peer Reviewed; English, French

**Web of science**

**TOPIC:** (exercis* OR endurance OR aerobic* OR "resistance activit*" OR "resistance train*" OR "Muscl* exercis*" OR "Muscl* strenght*" OR Sport OR "Physical activit*" OR "physical train*" OR "physical therap*" OR "Physical rehabili*" OR Relaxation OR Flexibility OR Stretch* OR walk OR yoga) *AND* **TOPIC:** (motiv* OR barrier* OR facilitator* OR obstacle* OR preference*)

*AND* **TOPIC:** (obes*)

Refined by: LANGUAGES: ( ENGLISH OR FRENCH ) AND DOCUMENT TYPES: ( ARTICLE ) AND RESEARCH AREAS: ( HEALTH CARE SCIENCES SERVICES OR OBSTETRICS GYNECOLOGY OR PUBLIC ENVIRONMENTAL OCCUPATIONAL HEALTH OR REPRODUCTIVE BIOLOGY OR ENDOCRINOLOGY METABOLISM OR SOCIAL SCIENCES OTHER TOPICS OR NUTRITION DIETETICS OR SOCIOLOGY OR BEHAVIORAL SCIENCES OR MEDICAL LABORATORY TECHNOLOGY OR PSYCHOLOGY OR SOCIAL WORK OR RESEARCH EXPERIMENTAL MEDICINE OR ORTHOPEDICS OR GENERAL INTERNAL MEDICINE OR SPORT SCIENCES OR SURGERY OR FOOD SCIENCE TECHNOLOGY OR LIFE SCIENCES BIOMEDICINE OTHER TOPICS OR NEUROSCIENCES NEUROLOGY OR COMMUNICATION OR PSYCHIATRY OR ENVIRONMENTAL SCIENCES ECOLOGY OR NURSING OR REHABILITATION OR CARDIOVASCULAR SYSTEM CARDIOLOGY OR RESPIRATORY SYSTEM OR EDUCATION EDUCATIONAL RESEARCH )

**PROQUEST - Physical Education Index**

SU("Exercise") AND SU("Obesity") AND (all(barrier*) OR all(preference*) OR all("facilitator*") OR all("obstacle*") OR all ("Motiv*"))
Limits : peer reviewed + English + humans
