# Supplementary material for: Physical activity motives, barriers, and preferences in people with obesity: A systematic review
Source: PLoS One. 2021 Jun 23;16(6):e0253114. doi: 10.1371/journal.pone.0253114 (PMC8221526; doi:10.1371/journal.pone.0253114)
Supplement: S1 Table — (DOCX) [file pone.0253114.s003.docx]

|  | | | | | | |
| --- | --- | --- | --- | --- | --- | --- |
| **Quantitative studies** | | | | | | |
|  | 1. Is the sampling strategy relevant to address the research question? | 2. Is the sample representative of the target population? | 3. Are the measurements appropriate? | 4. Is the risk of nonresponse bias low? |  | Total score % |
| Ashton [55] | No | No | Yes | Can't tell |  | 25 |
| Masterson [56] | No | No | Yes | Can't tell |  | 25 |
| Egan [57] | No | Can't tell | No | Can't tell |  | 0 |
| Genkinger [58] | No | No | Yes | Yes |  | 50 |
| James [59] | No | No | Yes | Yes |  | 50 |
| Labrunee [60] | No | No | No | Can't tell |  | 0 |
| Napolitano [61] | No | No | Yes | Can't tell |  | 25 |
| Rimmer [62] | No | No | Yes | Can't tell |  | 25 |
| Rye [63] | Yes | Yes | No | Can't tell |  | 50 |
| Skov-Ettrup [64] | Yes | No | No | No |  | 25 |
| Stankevitz [65] | No | No | Yes | No |  | 25 |
| Burton [66] | Yes | Yes | No | Yes |  | 75 |
| Short [67] | Yes | No | Yes | No |  | 50 |
| Borodulin [68] | Yes | Yes | No | Yes |  | 75 |
| **Qualitative studies** | | | | | | |
|  | 1. Is the qualitative approach appropriate to answer the research question? | 2. Are the qualitative data collection methods adequate to address the research question? | 3. Are the findings adequately derived from the data? | 4. Is the interpretation of results sufficiently substantiated by data? | 5. Is there coherence between qualitative data sources, collection, analysis and interpretation? |  |
| Bowen [69] | Yes | Yes | Yes | Yes | Yes | 100 |
| Coe [70] | Yes | Yes | Yes | Yes | Yes | 100 |
| Danielsen [71] | Yes | Yes | Yes | Yes | Yes | 100 |
| Guess [72] | Yes | Yes | Yes | No | No | 60 |
| Igelström [73] | Yes | Yes | Yes | Yes | Yes | 100 |
| Joseph [74] | Yes | Yes | Yes | Yes | Yes | 100 |
| Lidegaard [75] | Yes | Yes | Yes | Yes | Yes | 100 |
| Piana [76] | Yes | Yes | Yes | Yes | Yes | 100 |
| Lewis [78] | No | Yes | Yes | Yes | No | 60 |
| Groven [77] | Yes | Yes | Yes | Yes | Yes | 100 |
| **Mixed studies** | | | | | | |
|  | 1. Is there an adequate rationale for using a mixed methods design to address the research question? | 2. Are the different components of the study effectively integrated to answer the research question? | 3. Are the outputs of the integration of qualitative and quantitative components adequately interpreted? | 4. Are divergences and inconsistencies between quantitative and qualitative results adequately addressed? | 5. Do the different components of the study adhere to the quality criteria of each tradition of the methods involved? |  |
| Adachi-Mejia [79] | Yes | Yes | Yes | Yes | No | 80 |
| Lattimore [80] | Yes | Yes | Yes | Yes | No | 80 |
| Leone [81] | Yes | Yes | Yes | Yes | No | 80 |
