# Supplementary material for: Physical activity motives, barriers, and preferences in people with obesity: A systematic review
Source: PLoS One. 2021 Jun 23;16(6):e0253114. doi: 10.1371/journal.pone.0253114 (PMC8221526; doi:10.1371/journal.pone.0253114)
Supplement: S2 Table — (DOCX) [file pone.0253114.s004.docx]

|  | **References** | **Survey items/question OR**  **Second-order construct: “*First-order construct*”** | **Average response** | **Scores** |
| --- | --- | --- | --- | --- |
| **Physical factors** | | | | |
| ***Weight management*** | Ashton [55] | Lose weight | 46.4 % reported as key motivator | 3 |
|  | Skov-Ettrup [64] | Lose weight |  | 2 |
|  | Leone [81] | Weight management, Weight-loss may be the only benefit which is desirable enough to motivate obese women to overcome their barriers to exercise*; “Losing weight, seeing the numbers go down on the scale, feeling as though my clothes are fitting better or I get into, you know my smaller size clothes and that sort of thing that motivates me to work out, to be more purposeful in doing physical activity”* | Most common perceived | 3 |
|  | Guess [72] | Weight loss as a primary motivation*: “Right, and when you’re looking at the scale, each time maybe I go to the gym, you lose 1 pounds 2 pounds, so I think afterwards, I am losing, so I think to myself I need to keep going, it’s motivating to me.”; “Yes, always 3 times a week, after working, losing the weight, it motivates you, makes you feel you want to do a little bit more”; “I mean, does the person really come to the doctor and say I want to exercise because I want to be healthy or send me to the gym because I want to be healthy? No! They go on exercise and diet programs because they want to lose weight”; "Nothing is stopping me at the moment once I start to lose a little bit of weight I start to feel motivated”* | Primary motivation for nearly all women | 3 |
|  | Danielsen [71] | Persistence in weight regulation grounded in a fundamental fear of gaining weight. What they feared the most was to “go back to where I was.” |  | 2 |
|  | Igelström [73] | Desire for a change: *“I want to lose weight”; “I am fed up with being in this situation”* |  | 2 |
|  | Groven [77] | Weight reduction is seemed by women as essential; *‘‘You actually exercise your entire body. You go through every muscle in your body. I think that is very important, especially when it comes to burning fat. You have to push your body in order to burn fat and lose weight. ”.* |  | 2 |
|  | Lewis [78] | To lose weight to becoming “fit,” “healthy,” and “athletic” rather than “thin”. *"Men don’t want to be thin. Our goal is to have low body fat, to be muscular. The ideal body shape for men is quite a muscular one in men’s minds."* | Described by many men | 3 |
| ***Energy/physical fitness/sport performance*** | Ashton [55] | Improve fitness, improve muscle mass, improve sporting performance |  | 2 |
|  | Skov-Ettrup [64] | Get or stay fit |  | 2 |
|  | Bowen [69] | To increase energy: *“When you do it [physical activity], you do feel better. I’m not tired I just feel good”; “I’m not as tired as I used to be. And I just feel good. ”* | One of the most expressed motivational factors. | 3 |
|  | Danielsen [71] | *“Now I notice that I get a lot more energy from being active. Therefore, if I’m tired, going to the gym or going for a walk or doing something [physical activity] is useful for me”.*  Change in lifestyle and self-perception due to improved physical fitness*: “Before I was a spectator in my own life, now I’m participating.”* |  | 2 |
|  | Joseph [74] | Increase energy: *“Have more energy”. “Being able to maybe keep up with family – with my son and my husband – and being able to do those things and not be exhausted or not sit out the ride or sit out the trip for whatever reason”* |  | 2 |
|  | Lidegaard [75] | Give energy to the rest of everyday life; *When you do it [physical activity], you do feel better. I’m not tired I just feel good”. “I’m not as tired as I used to be. And I just feel good.”* |  | 2 |
| ***Health*** | Ashton [55] | Improve overall health, to live longer |  | 2 |
|  | Bowen [69] | To decrease pain and stiffness | The most commonly expressed motivational factors | 3 |
|  | Joseph [74] | Health outcomes; Reduced risk for chronic disease *“Ultimately it’s just increased health. Be healthier, you’ll feel better, have more energy. Better response to stress, all those kinds of things”* | Mentioned in all focus group | 3 |
|  | Lidegaard [75] | To maintain or improve health; Increased motivation if visible improvement in health parameters; Reviewing/reaching health goals *"One of the good things about diabetes is that you can measure pretty quickly if what you’re doing is the right thing [. . . ] instead of waiting for the rather long-term effect of exercise on my weight. The immediate responses is really motivating to me. "* |  | 2 |
| **Psychological factors** | | | |  |
| ***Fear to die*** | Igelström [73] | Threat or coercion; Direct advice from a health care provider *“If someone told me you’re going to die tomorrow if you don’t exercise, then of course I would be more physically active”* | Some participants | 1 |
|  | Joseph [74] | Reduced risk of chronic conditions*; “For me, the motivating factor is I want to live. You know. Straight up, I want to live…you see people who are passing away at your age, I guess. And, you know, because of heart attacks or, you know, things, diabetes or whatever these things”* | Mentioned in all focus group | 3 |
| ***Well-being*** | Ashton [55] | Improve mental health and well-being, improve sleeping patterns, |  | 2 |
|  | Danielsen [71] | Feeling of vigor and better mood | Important incentive for PA | 3 |
|  | Lidegaard [75] | Good sense in the body, sense of well being |  | 2 |
| ***Appearance*** | Ashton [55] | Improve body image, attract a partner |  | 2 |
|  | Joseph [74] | To improve physical appearance*: “My motivation would be clothes, actually. Yeah, and not having to buy plus size clothes, and not having to spend ridiculously large amounts of money for clothing.”* | Mentioned by some women | 1 |
| ***Pleasure*** | Ashton [55] | Enjoyment |  | 2 |
|  | Skov-Ettrup [64] | Have fun |  | 2 |
| ***Stress management*** | Skov-Ettrup [64] | Unwind |  | 2 |
|  | Lidegaard [75] | An opportunity to reduce stress and recharge |  | 2 |
|  | Bowen [69] | *“It has helped me with stress”* |  | 2 |
| ***Self-care*** | Joseph [74] | Self-care*: “If we love ourselves, then we’ll do this for ourselves.”* |  | 2 |
|  | Lidegaard [75] | To have time for yourself |  | 2 |
| **Socio-ecological factors** | | | | |
| ***Independence*** | Bowen [69] | To maintain independence with Daily Living and Instrumental. Activities of Daily Living. Independence was a factor that was valued among all the women and a motivator to be physically active because it played a vital role in their health. | All women | 3 |
| ***Role model*** | Joseph [74]  . | Being a good role model to others, particularly their family: *“I have children. They’re teenagers, and I’m trying to break out of the statistics category regarding African-American women [referring to cardiometabolic health disparities]”. “I would say setting a good example for my children is another motivating factor.”* | Several women | 1 |
| ***Social support*** | Asthon [55] | Social influences |  | 2 |
|  | Skov-Ettrup [64] | Socialize |  | 2 |
|  | Lidegaard [75] | Being physically active with others; Social interaction “*I think it’s a good idea to work out with other people. I like that a lot because of the social aspect. . . I play football, and we’ve got a tradition of a “third half”, with food and having fun. That’s really good [. . .] all that stuff with the locker room. I think that’s important for a lot of people.”*  Commitment; Sharing good experiences; Friendly competition; Mutual inspiration: *“I do fitness twice a week with other people. Well, I do it that way because if I was to do it alone, I’d just procrastinate [. . .] I just have to oblige myself to do it with other people; otherwise, I’d never do it.”* | Emphasized by many participants | 3 |
|  | Groven [77] | Motivated by treatment group: *“The others stayed in the program, which made me hang in as well. It sort of motivated me, seeing the other participants pushing themselves”.*  Motivated by a physiotherapist: *‘‘She [physiotherapist] is direct whether it can hurt us or please us. "She tells us how to do things better. But I think it important that she also tells me that I can do better. I like the way she pushed us,’*  They emphasized that exercising together with persons in the same situation as themselves [treatment group for organized for patients with obesity problem] made them feel more recognized and accepted. Sense of belonging. Treatment group provides a common ground in which the women can feel equal, regardless of their body weight. |  | 2 |
|  | Lewis [78] | More motivated to engage in physical activity if it involved family members or friends: *“You’d never go and run around the track 4 times a week, but you might if you knew it was a good time and you’d meet other people who were all doing the same.”* |  | 2 |
|  | Danielsen [71] | Challenged by others in a group class to “try things”: *"It was motivating. Because most of them were “in the same boat” as me, they wanted to exercise and got a “kick” from it. They wanted to exercise more and get involved and engaged, so we motivated and helped each other,… "*  Share positive and negative experiences *‘‘Here I can meet others with the same problems as myself. For everyone have problems due to their weight, right. And we share the same problems,… We understand each other"*  Being in the same boat*: “We looked a little similar, everything was in a way very safe”; “It was okay to breathe and wheeze and it was okay to [let the fat] wiggle and all this, you know, when we exercised.”* | Several participants | 1 |
