# Supplementary material for: Physical activity motives, barriers, and preferences in people with obesity: A systematic review
Source: PLoS One. 2021 Jun 23;16(6):e0253114. doi: 10.1371/journal.pone.0253114 (PMC8221526; doi:10.1371/journal.pone.0253114)
Supplement: S3 Table — (DOCX) [file pone.0253114.s005.docx]

|  | **References** | **Survey items/question OR**  **Second-order construct: “*First-order construct*”** | **Average response** | **Scores** |
| --- | --- | --- | --- | --- |
| **Physical factors** | | | |  |
| ***Poor health*** | Napolitano [61] | Poor health | 1.45 /5 for agreement | 1 |
|  | Rimmer [62] | Health concerns prevent me from exercising | 39% agreed | 2 |
|  | Adachi-Mejia [79] | Lack of general good health | As weight 🡕, this item 🡕 in frequency | 2 |
|  |  | Arthritis; Hip issues; Chronic illness; Foot issues; Knee issues; Health issues; Ankle injury; Asthma; Back issues; Migraines; Diabetes; Smocking. | Knee issues were described as a barrier in all weight classes. Asthma was mentioned as barriers in all obese classes. Participants described more types of physical barriers as weight class increased |  |
|  | Coe [70] | Health concerns  Physical limitations | Mentioned by all members  Mentioned by all male participants | 3 |
|  | Guess [72] | *“[Physical activity] is not really for someone like me with my health conditions”.* |  | 2 |
|  | Igelström [73] | Medical problems |  | 2 |
|  | Lattimore [80] | Health: “*Arthritis, fibromyalgia, muscular, degeneration, heart conditions, and surgeries”* | 32.3% | 2 |
|  | Leone [81] | Chronic condition: “*I can’t do it anymore, because I can’t walk like that anymore ‘cause my blood pressure spikes”*  Injury or surgery | Second most common barrier to exercise. | 3 |
|  | Lidegaard [75] | Poor physical conditions: “*I can’t ride a bicycle and walk as I used to because of problems with my legs, right?”* | Substantial barrier | 3 |
| ***Pain/ physical discomfort*** | Labrunee [60] | Pain following PA | very frequent [>60%] | 3 |
|  | Napolitano [61] | Minor aches and pain | 2.13/ 5 for agreement | 2 |
|  | Egan [57] . | Physical discomfort | 23.4 % reported as a major barrier [# 1 barrier] | 3 |
|  | Rimmer [62] | Pain prevents me from exercising | 64% agreed | 3 |
|  | Stankevitz [65] | Pain or discomfort | 2.7/5 for frequency | 1.5 |
|  |  | Feeling nauseated | 1.5/5 for frequency |  |
|  | Adachi-Mejia [79] | Pain | Mentioned as barrier in all obese classes [I, II and III] | 2 |
|  | Bowen [69] | Chronic pain: “*My legs hurt when I walk and [with] standing; when I stand too long, it’s my back” “I don’t participate in the exercise. I used to but my back bother me now… So I don’t exercise. I can’t go through that exercise [at the gym]”* | Mentioned by many participants and as a significant barrier. | 3 |
|  | Leone [81] | Physically uncomfortable while exercising |  | 2 |
|  | Danielsen [71] | Exercise is painful; Pain related to limited mobility: *“I’ve never had so much pain”* | Experienced by all participants. | 3 |
|  | Igelström [73] | Pain as a side effect of physical activity; Bodily sensations: *“Unpleasant sensations from the heart while exercising”* |  | 2 |
|  | Lidegaard [75] | Pain: *“I’m pretty sure I do get the exercise, but I should do more of it. But when I do more of it, well then it’s hard on my knees and back.”* | 50% | 3 |
|  | Piana [76] | *“Muscle pain” “injuries, accidents”* |  | 2 |
|  | Groven [77] | Pain: *‘‘Pain in my knees, in my back, in my groin. I really felt like a failure.”* | Many | 2 |
| ***Weight*** | Napolitano [61] | I am too overweight | 2.74/5 for agreement | 2 |
|  | Leone [81] | My current weight makes it difficult for me to exercise | 46.5% agreed | 2.5 |
|  |  | Weight: *“…the weight goes down and then I feel better about exercising, because who wants to be, you know, big, fat, tight clothes, that kind of thing, getting out of breather earlier or something like that, although my breath really is pretty good. ”* | Weight-related barrier was the first common theme |  |
|  | Lattimore [80] | *Being overweight [both currently and for life]* |  | 2 |
|  | Danielsen [71] | Living a heavy body: *“I couldn’t do anything ”* |  | 2 |
|  | Guess [72] | Weight | Majority of participants | 3 |
|  | Adachi-Mejia [79] | Weight | Mentioned as barriers in all obese classes. | 2 |
|  | Piana [76] | Weight: *“Difficulty of movement caused by obesity” “Difficulty in tying my shoe-laces and/or standing up”* |  | 2 |
| ***Fatigue/lack of energy*** | Napolitano [61] | Lack of energy | 3.47/5 for agreement | 3 |
|  |  | I am usually too tired to exercise | 3.11 /5 for agreement |  |
|  | Rimmer [62] | Lack of energy | 36% agreed | 2 |
|  | Egan [57] | Too tired to exercise | 15.9 % reported as a major barrier | 2 |
|  | Genkinger [58] | Too fatigued by exercise | 13% strongly agreed or agreed | 1.5 |
|  |  | Too tired | 37% strongly agreed or agreed |  |
|  | Stankevitz [65] | Fatigue [lack of energy] | 3.3/5 for frequency | 3 |
|  | Leone [81] | I don’t have the energy to exercise | 57.8% agreed | 3 |
|  | Adachi-Mejia [79] | Lack of energy |  | 2 |
|  |  | Low energy; Sleep issues; Tiredness | Low energy was mentioned in two obese classes [I and II]; Sleep issues in one obese class [II]; Tiredness in all obese classes. |  |
|  | Joseph [74] | “Once I get home, I’m done. I don’t want to do nothing.” | Commonly reported barrier | 3 |
|  | Lattimore [80] | Lack of energy | 15% | 1 |
|  | Bowen [69] | Fatigue; Fatigue contributed to a person’s inability to perform regular physical activity and leads to a sedentary lifestyle | Mentioned by two women [22%] | 1 |
|  | Piana [76] | Fatigue*: “Fatigue”* |  | 2 |
| ***Poor physical fitness*** | Labrunee [60] | My poor effort capacity | very frequent [>60%] | 3 |
|  |  | The bad tolerance to efforts [dyspnea, muscular fatigue, feeling of discomfort, pain] | very frequent [>60%] |  |
|  | Napolitano [61] | Physical activity is hard work | 2.38 /5 for agreement | 2 |
|  | Adachi-Mejia [79] | Out of shape; Exhaustion | Out of shape was mentioned in two obese classes [I and III]; Exhaustion in one obese class [I] | 2 |
|  | Igelström [73] | Exhaustion: “*Exhaustion* “ |  | 2 |
| ***Aging*** | Rimmer [62] | Too old to exercise | 6% agreed | 1 |
|  | Bowen [69] | Aging; Exercise becomes more difficult with aging: *“The older I get I can’t run or jump like I use to”.* | Mentioned by 3 participants [33%] | 2 |
| **Psychological factors** | | | |  |
| ***Fear*** | Adachi-Mejia [79] | Fear of injury | As weight ⭧, item ⭧ in frequency | 2 |
|  | Rimmer [62] | Exercise will make my condition worse | 6% agreed | 1 |
|  | Napolitano [61]. | Fear of injury | 1.45 /5 for agreement | 1 |
|  | Stankevitz [65] | Fear of injury | 2.0/5 for frequency | 2 |
|  | Ashton [55] | Injury |  | 2 |
|  | Labrunee [60] | Doing a physical activity frightens me because of: 1- The risk of hypoglycemia; 2- The risk of not controlling my diabetes 3- The risk to get tired; 4- The risk to be injured; 5- The risk of having a cardiac problem | < 40% | 2 |
|  | Danielsen [71] | Fear of get injured: *“I was sure I would die”.* |  | 2 |
|  | Guess [72] | Fear of get injured: *“I don’t want to do myself damage”*  *“Due to my knee at this moment in time I do feel like that [a need for specialist advice] would maybe be the case”.* |  | 2 |
|  | Piana [76] | Fear of get injured; Fear of not succeeding: *“Fear of failing”* |  | 2 |
| ***Lack of self-discipline or motivation*** | Leone [81] | I usually only exercise if I am trying to lose weight | 42.4% agreed | 2 |
|  | Stankevitz [65] | Lack of self-discipline | 3.5/5 for frequency | 3 |
|  |  | Procrastination | 3.3/5 for frequency |  |
|  | Ashton [55] | Lack of motivation |  | 2 |
|  | Borodulin [68] | Lack of motivation |  | 2 |
|  | Labrunee [60] | Low motivation | very frequent [>60%] | 3 |
|  | Napolitano [61] | Lack of self-discipline or willpower | 4.27/5 for agreement | 3 |
|  | Rye [63] | Lack of willpower | 54.5% agreed | 3 |
|  | James [59] | I don't have the willpower to exercise | Obese class 1 and 2 reported stronger levels of agreement with this barrier than other weight group | 2 |
|  | Genkinger [58] | Lack of motivation | 63% strongly agreed or agreed | 3 |
|  | Rimmer [62] | Lack of motivation | 30% agreed | 2 |
|  | Adachi-Mejia [79] | Self-discipline | Most frequently mentioned barrier among people with obesity | 2.5 |
|  |  | Lack of motivation; In a rut; Don’t see results | Lack of motivation was mentioned for four out of five weight classes; In a rut in one obese class [II]; Don`t see results in one obese class [III] |  |
|  | Piana [76] | Lack of motivation, Too many excuses; Lack of personal incentives  : *“Not achieving the target” “Why am I trying?”* “Excuses for not doing it” “It is hard to get going” |  | 2 |
|  | Lattimore [80] | Lack of motivation; motivation | 28.3% and 41.6% | 2 |
|  | Lidegaard [75] | Broken exercise routine and difficulty to start exercising again: *“I just didn’t do it as a daily routine [. . .] It took several months to get it running again as a routine.”* | Minor topic | 1 |
|  | Joseph [74] | Don`t see results: *“Sometimes all the goals make you feel like the little bit you do isn’t worth it.”* |  | 2 |
| ***Lack of interest or enjoyment*** | Genkinger [58] | Exercise is boring | 18% strongly agreed or agreed | 1 |
|  | Egan [57] | Exercise is too boring | 20.7 % reported as a major barrier [# 2 barrier] | 2 |
|  |  | Dislike of gym | 9.7 % reported as a major barrier |  |
|  | Napolitano [61] | PA is too boring | 2.26 /5 for agreement | 2.3 |
|  |  | Lack of interest in PA | 3.13 /5 for agreement |  |
|  |  | I do not enjoy PA | 2.66 /5 for agreement |  |
|  | Rimmer [62] | Lack of interest | 9% agreed | 1 |
|  |  | PA is boring or monotonous | 24% agreed |  |
|  |  | Satisfied with physical appearance | 12% agreed |  |
|  |  | Exercise is too difficult | 18% agreed |  |
|  | Stankevitz [65] | Exercise is not in routine | 3.2/5 for frequency | 2.4 |
|  |  | Lack of interest in exercise | 2.9/5 for frequency |  |
|  |  | Lack of enjoyment from exercise | 2.7/5 for frequency |  |
|  |  | Exercise was boring | 2.5/5 for frequency |  |
|  |  | Exercise is not a priority | 3.0/5 for frequency |  |
|  | Rye [63] | Don’t need more | 4.6 % agreed | 1 |
|  | Labrunee [60] | The lack of desire | very frequent [>60%] | 3 |
|  | Adachi-Mejia [79] | Prefer to do other things; Not fun; Dislike exercise; Not thinking about doing exercise; Sedentary activities | Prefer to do other things was mentioned in one obese class [I]; Not fun in one obese class [I]; Dislike was mentioned in one obese class [II]; Not thinking about doing exercise in one obese class [I]; Sedentary activities in one obese class [III]. | 2 |
|  | Lattimore [80] | Lack of interest | 5.7% | 1 |
|  | Piana [76] | Resistance to practice unpleasant activities: *“I hate running, and doing physical activity is unpleasant".* |  | 2 |
| ***Mental health concerns*** | Egan [57] | Too depressed to exercise | 5.5 % reported as a major barrier | 1 |
|  | Adachi-Mejia [79] | Stress; Depression; Mood; Lazy; Procrastination; Impatience; Sadness | Depression was mentioned as a barrier in all three obese classes. Stress and mood were mentioned in one obese class [I]. Lazy was mentioned in two obese classes [II, III], Procrastination was mentioned in one obese class [II]. Impatience was mentioned was mentioned in one obese class [III] | 2 |
|  | Bowen [69] | Laziness | Mentioned by 2 participants [22%] | 1 |
|  | Igelström [71] | Psychological distress | Some participants | 1 |
| ***Negative past experiences*** | Egan [57] | Negative past experience of exercise | 4.1 % reported as a major barrier | 1 |
|  | Napolitano [61] | I hate to fail so I do not try | 2.13/5 for agreement | 2 |
|  | Igelström [73] | Negative past experience of exercise | Some participants | 1 |
|  | Joseph [74] | Being forced to perform activities they did not want to perform; Being uncomfortable with PA due to early onset of physical development; Embarrassment and teasing from other children; Hiring a personal trainer and/or engaging in structured exercise classes that were too intense or beyond their skill level.  *“My personal trainer was negative in his approach” “I signed up for a free class. It was a Cross-Fit class [….]* *the next day I felt like I was hit by a truck, and I didn’t go back. ”* | A few women | 1 |
|  | Lattimore [80] | Negative exercise perceptions or  Experience | 4% | 1 |
| ***Self-conscious*** | Egan [57] | Embarrassed about physical appearance | 4.1 % reported as a major barrier | 1 |
|  | Napolitano [61] | Self-conscious about my look when I do activities | 2.6 /5 for agreement | 2 |
|  | Labrunee [60] | Doing a physical activity frightens me because of: 6- The others’ look | < 40% | 2 |
|  | James [59] | I am uncomfortable with how I look while exercising or while wearing exercise clothing | Obese class 1 and 2 reported stronger levels of agreement with this barrier than other weight groups | 2 |
|  | Rimmer [62] | Feel uncomfortable exercising in a fitness center | 30% agreed | 2 |
|  | Ashton [55] | Intimidation/embarrassment |  | 2 |
|  | Leone [81] | I am uncomfortable with how I look while exercising or while wearing exercise clothing | 44.4 % agreed | 1.5 |
|  |  | Uncomfortable with appearance while exercising: *“I want to exercise by myself, but the only reason I don’t want to join a club, or anything like that is because everybody in there is like a size 2”* | 15.8% |  |
|  | Guess [72] | *“I am too self-conscious to go to the gym with all these skinny little women”.* |  | 2 |
|  | Danielsen [71] | Not fitting in to typical exercise contexts: *“As when you enter Elixia [national fitness center] with 140 kg and the others are highly trained people, then you feel a bit like that, you know what I mean?” “Feeling of bodily “otherness.”* | Some participants. | 1 |
| ***Lack of skill or confidence*** | Napolitano [61] | Lack of skill | 2.14 /5 for agreement | 2 |
|  | Ashton [55] | Lack of skills/knowledge |  | 2 |
|  | Stankevitz [65] | Lack of skill | 2.2/5 for frequency | 2 |
|  | Adachi-Mejia [79] | Lacking coordination  Self-perception as non-physical | Lacking coordination and self-perception as non-physical were mentioned as barrier in one obese class [II] | 2 |
|  | Danielsen [71] | Feeling of not being able to do anything: *“I couldn’t do anything.”* | Prominent theme | 3 |
|  | Guess [72] | Lack of confidence related to ability and perceived ability. *“I wouldn’t feel comfortable doing it you know in a public place you know, obviously you have limitations.”* | Many participants | 3 |
|  | Igelström [73] | Doubt if they will be able to obtain the benefits | Some participants | 1 |
|  | Joseph [74] | Participants reported their own self as a primary source of discouragement: *“I don’t necessarily have anyone who’s not supportive, but I know I can be my own worst critic.”* |  | 2 |
| ***Beliefs*** | Rimmer [62] | Exercise will not improve my condition | 9% agreed | 1 |
|  | Ashton [55] | Exercising is not a masculine/bloke thing to do |  | 2 |
|  | Guess [72] | Resistance exercises as a masculine activity; *“I don`t want build muscles. To me it’s like a man’s sport. ”* | Amongst all the participants | 3 |
|  | Joseph [74] | Participants belief that the racial history contributes to health benefits related to PA: *“It’s [referring the PA for health benefits] really tailored to the individual because there’s, ‘What’s your racial history?’ And not just what you might think you see, but what’s your history because that can contribute to your health benefits.”* | Few participants | 1 |
|  | Leone [81] | Only women who are trying to lose weight need to exercise: *“I have never had to exercise in my young life to stay in shape.”*  *“I think weight gain does drive exercise. If I could weigh 110 pounds and never exercise, I’d be very happy.”* |  | 2 |
|  | Lewis [78] | Being too concern about health is not masculine |  | 2 |
| **Socio-ecological factors** | | | |  |
| ***Lack of time*** | Napolitano [61] | Lack of time | 3.43 /5 for agreement | 3 |
|  | Rye [63] | Lack of time | 25.7% agreed | 2 |
|  | Rimmer [62] | Lack of time | 15% agreed | 1 |
|  | Egan [57] | No time to exercise | 20 % reported as a major barrier [# 3 barrier] | 3 |
|  | Ashton [55] | Lack of time to exercise because of busy lifetime |  | 2 |
|  | Borodulin [68] | Lack of time |  | 2 |
|  | Genkinger [58] | No time to exercise | 50% strongly agreed or agreed | 2.3 |
|  |  | Time Inconvenient | 28% strongly agreed or agreed |  |
|  |  | Too busy to exercise | 47% strongly agreed or agreed |  |
|  | Masterson [56] | Hard to make time | 39 % agreed | 2.5 |
|  |  | Cannot make time | 81.6 % agreed |  |
|  | Stankevitz [65] | Inconvenient exercise schedule | 3.1/5 for frequency | 3 |
|  |  | Lack of time | 3.4/5 for frequency |  |
|  | Adachi-Mejia [79] | Lack of time |  | 2 |
|  |  | Lacking time; Schedule | Lacking time was mentioned as barrier in all weight classes; Schedule in one obese class [I]. |  |
|  | Lattimore [80] | General lack of time. time | 13.3% and 30.6% | 1.5 |
|  | Danielsen [71] | Conflict with other activities and social events; Exercise as time-consuming. *“You have to give up other things in order to exercise.”* |  | 2 |
|  | Igelström [73] | Running out of time: *“I already have too much to do and adding physical activity will only stress me further. ”* |  | 2 |
| ***Too many obligations*** | Genkinger [58] | Too many family obligations | 29% strongly agreed or agreed | 2 |
|  | Stankevitz [65] | Family responsibilities | 3.3/5 for frequency | 3 |
|  | Napolitano [61] | Family demands | 2.77 /5 for agreement | 2 |
|  |  | Social demands | 2.26 /5 for agreement |  |
|  |  | Work demands | 2.70 /5 for agreement |  |
|  | Rimmer [62] | Job responsibilities prevent me from exercising | 3% agreed | 1 |
|  |  | Family responsibilities prevent me from exercising | 21% agreed |  |
|  | Adachi-Mejia [79] | Obligations; Caregiving duties; Family demands; Home life; Work; Sedentary desk jobs | Caregiving duties and family in one obese classe [I]; Home life in two obese classes [I and II]; Work in all five of the weight classes; Sedentary desk jobs in one obese class [II]. | 2 |
|  | Lewis [78] | Long working hours; Desk jobs | Extreme barriers to physical activity | 2.75 |
|  |  | Work demands; Family commitments *“[…] when I got married I basically stopped being active to spend more time with the missus”.* | [n=20, 56%]  [n=11, 31%] |  |
|  | Lattimore [80] | Work and family constraints | 7.5 and 15% | 1 |
|  | Lidegaard [75] | Other priorities and interests: *“I just don’t have time for it. Well, when I get home, I just get sucked into family life.”; “I always have so many things to do at home that are a thousand times more important than getting out and exercising. ”* | All participants | 3 |
| ***Lack of social support*** | Rye [63] | Lack of support | 52.6 % agreed | 3 |
|  | Ashton [55] | Social influences [e.g., because your partner, family, or friends are NOT active] |  | 2 |
|  | Egan [57] | Nobody to exercise with | 3.4 % reported as a major barrier | 1 |
|  |  | Lack of support from friends and family | 0.7 % reported as a major barrier |  |
|  | Napolitano [61] | I do not have anyone to do physical activities with me | 2.97 /5 for agreement | 2 |
|  | Stankevitz [65] | Lack of company | 2.7/5 for frequency | 2 |
|  | Leone [81] | I don’t have any one to exercise with | 48.5 % agreed | 2 |
|  | Borodulin [68] | Lack of companion for activity |  | 2 |
|  | Rimmer [62] | Lack of friends/family support | 6% agreed | 1 |
|  | Adachi-Mejia [79] | Lack of compagny |  | 2 |
|  |  | Being alone; No one to exercise with | Being alone was mentioned in two obese classes [II and III]; No one to exercise in one obese class [I]. |  |
|  | Igelström [73] | Lack of family and friends support; Lack of health care services support | Majority | 3 |
|  | Coe [70] | Lack of interpersonal support; Lack of community support; Feeling isolated and misunderstood. | Several male and female participants. | 1 |
|  | Piana [76] | *“Loneliness”* |  | 2 |
|  | Lattimore [80] | Lack of an exercise partner; Lack of social support; Lack of accountability person | 0.6, 1.2 and 0.6% | 1 |
| ***Cost*** | Masterson [56] | Cost | 50.4 % agreed | 3 |
|  | Rimmer [62] | Cost of program | 67% | 3 |
|  | Stankevitz [65] | Cost of exercising | 2.8/5 for frequency | 2 |
|  | Napolitano [61] | Lack of money | 1.85 /5 for agreement | 1 |
|  | Rye [63] | Lack of money | 37% agreed | 2 |
|  | Egan [57] | Too expensive to exercise | 4.8 % reported as a major barrier | 1 |
|  | Ashton [55] | High cost of equipment/facilities |  | 2 |
|  | Borodulin [68] | High expenses |  | 2 |
|  | Coe [70] | Low income and gyms/exercise classes costs | Majority of women and one of the most significant barriers | 3 |
|  | Igelström [73] | Costs |  | 2 |
|  | Lewis [78] | Affordability of living a healthy lifestyle: “*Exercise equipment and gym memberships are very expensive, and I think manufacturers, particularly of exercise equipment, put their prices up and try to capitalize on people who may be desperate to lose weight and in a desperate bid to lose weight will pay inflated prices for products.”* | 16.6% | 1 |
| ***Lack of access to equipment, facilities or professional*** | Masterson [56] | Accessible place | 30.9 % agreed | 2 |
|  | Egan [57] | Transport issues | 1.4 % reported as a major barrier | 1 |
|  | Ashton [55] | Lack of facilities |  | 2 |
|  | Rimmer [62] | Lack of accessible facilities | 46% agreed | 2 |
|  |  | Lack of personal care attendant | 30% agreed |  |
|  |  | Lack of transportation | 49% agreed |  |
|  | Napolitano [61] | Lack of convenient place to do physical activity | 2.30 /5 for agreement | 1.5 |
|  |  | Lack of equipment | 1.98 /5 for agreement |  |
|  | Rye [63] | Lack of place | 18 % agreed | 1 |
|  | Adachi-Mejia [79] | Lack of facilities |  | 2 |
|  | Leone [81] | I don’t have any place to exercise. | 11.1% agreed | 1 |
|  | Stankevitz [65] | Lack of equipment | 2.3/5 for frequency | 1.7 |
|  |  | No facilities or space to exercise | 2.1/5 for frequency |  |
|  |  | Lack of knowledgeable exercise staff | 1.8/5 for frequency |  |
|  | Coe [70] | Lack of health and wellness related programs; Recreation centers in neighborhood had closed down; Transportation [lack of a car] | Mentioned by one female participant  Female participants | 1 |
|  | Igelström [73] | Equipment |  | 2 |
|  | Lidegaard [75] | Lack of accessibility to local  exercise facilities: ‘For me there’s something with the distance. Well, if it’s too big, I just can’t fit it in.’  “I’ve read about the guy Jerry from Allerød who does interval walking […] I don’t know if there’s someone who does interval walking closer to where I live.” | Almost half the participants | 2 |
|  | Lattimore [80] | Accessibility concerns | 7.5% | 1 |
| ***Bad weather*** | Egan [57] | Weather prevents exercise | 11.7 % reported as a major barrier | 2 |
|  | Genkinger [58] | Weather | 19 % strongly agreed or agreed | 1 |
|  | Napolitano [61] | Weather is bad | 2.62 /5 for agreement | 2 |
|  | Stankevitz [65] | Bad weather | 2.5/5 for frequency | 2 |
|  | Ashton [55] | Weather [e.g., too hot or too cold to exercise] |  | 2 |
|  | Adachi-Mejia [79] | Humidity; Rainy days; Too hot outside; Too cold outside; Too sunny; Too dark outside | Humidity and rainy days were mentioned in one obese class [I]; The weather being too hot outside in three obese classes [I, II and III]; Too cold outside in one obese class [II]; Too sunny in two obese classes [I and III]; Too dark outside in one obese class [III]. | 2 |
|  | Igelström [73] | Weather |  | 2 |
|  | Joseph [74] . | Extreme heat during the summer months. |  | 2 |
|  | Lattimore [80] | Weather | 15.6% | 1 |
| ***Safety issues*** | Genkinger [58] | Concern about safety | 10 % strongly agreed or agreed | 1 |
|  | Egan [57] | Roads too dangerous to exercise | 2.8 % reported as a major barrier | 1 |
|  | Lattimore [80] | Transportation and safety | 3.4% | 1 |
| ***Lack of knowledge or information*** | Napolitano [61] | Lack of knowledge on how to do physical activity | 2.05 /5 for agreement | 2 |
|  | Ashton [55] | Lack of information on appropriate exercise routines |  | 2 |
|  | Rimmer [62] | Don’t know how to exercise | 46% agreed | 2 |
|  |  | Don’t know where to exercise | 39% agreed |  |
|  |  | Not aware of fitness center in the area | 46% agreed |  |
|  | Adachi-Mejia [79] | Lacking knowledge | Mentioned in 1 class of obese [II] | 1 |
|  | Lattimore [80] | Lack of exercise knowledge or experience | 4.6% | 1 |
|  | Guess [72] | Lack of knowledge of how to do physical activity and certain types of physical activity ; Lack of knowledge about where to do physical activity.  *“I don’t know which ones [exercises] would tone up my arms or which ones are best for me to build up my strength”.“Basically I asked at the local gym and my GP but neither of them knew where to go”.* | Many participants | 3 |
|  | Lidegaard [75] | Lack of knowledge about how to exercise or be physically active: *“I really don’t know what I should do.’ “If I were to take part in that kind of exercise, I’d make it a condition that there were some professionals who knew what it was all about when you’ve got the problems I do”* | Substantial barriers | 3 |
|  | Joseph [74] | Lack of information: *“I would need something to show me what exercises to do on Monday, which ones to do on Tuesday…”* |  | 2 |
| ***Stigma*** | Coe [70] | Stigma: *Experiencing negative comments from strangers* | Mentioned by female participants | 2 |
|  | Piana [76] | Negative experiences: “*During the secondary school I never did sports because I was ashamed of my body”"At school they called me "fatty" and let me off the activity. I felt excluded, discriminated against.”* | Markedly negative influence on the patients’ attitude towards it. | 2 |
|  | Groven [77] | The gaze of others: *‘‘Sometimes I felt sick before going to the fitness training. I couldn’t stand the idea of been stared at.’’ [gym] ‘‘It made me feel disgusting. They looked at me because I was fat.. What is she doing her with a body like that kind of look. It made me feel like an idiot.”* | Mainly barrier | 3 |
|  | Lewis [78] | Weight-based stigma: *“Doing any physical activity, it’s not something to look forward to, it’s putting myself out there to be ridiculed again”.*  *“Just walking into a gymnasium is hugely embarrassing. You may as well walk in there naked because everyone turns to you and looks at you and you can just about hear them going ‘oh yuc”* | Few participants | 1 |
